# THE GERMAN TRANSFER CLUSTER OF ACADEMIC NURSING INSTITUTIONS IN LONG-TERM CARE (TCALL)

**DOI:** 10.1093/geroni/igae098.0712

**Published:** 2024-12-31

**Authors:** Karin Wolf-Ostermann, Benedikt Preuss, Heinz Rothgang, Ingrid Darmann-Finck, Karsten Wolf, Claudia Stolle-Wahl, Matthias Zuendel

**Affiliations:** University of Bremen, Bremen, Bremen, Germany; Research Center on Inequality and Social Policy (SOCIUM), University of Bremen, Bremen, Bremen, Germany; Research Center on Inequality and Social Policy (SOCIUM), University of Bremen, Bremen, Bremen, Germany; Institute of Public Health and Nursing Research, University of Bremen, Bremen, Bremen, Germany; University of Bremen, Bremen, Bremen, Germany; City University of Applied Sciences Bremen, Bremen, Bremen, Germany; Integrated Health Campus Bremen, Bremen, Bremen, Germany

## Abstract

Considering the demographic shift, ensuring high-quality care emerges as a central future challenge in Germany. This can only be overcome by enhancing the attractiveness of the nursing profession through improved working conditions, introducing technological innovations comprehensively into long-term care, and adapting internal workflows through personnel and organizational development, as well as evolving educational content and structures accordingly. Currently in Germany such transfer and innovation structures are lacking. Therefore, TCALL establishes a sustainable framework to enable the implementation of technical and organizational innovations in regular operations similar to the Living Lab in Ageing and Long-Term Care (Netherlands) or Teaching Nursing Homes (US). This approach facilitates a direct pathway for research findings to transfer from academia through education into healthcare practice, with reciprocal influences from healthcare practice back into research and education. We will present the concept of TCALL which is up to now implemented in three nursing homes in the federal state of Bremen, Germany and will be further developed into a cluster of academic nursing institutions through the addition of personnel and the establishment of a decentralized learning infrastructure. TCALL thus constitutes a conceptual space for innovative transfer activities and simultaneously provides a permanent physical environment for recursive innovation development, testing, and implementation. This structure enables the close integration of research, education, and practice, contributing directly to the rapid and process-oriented development of the state-of-the-art through innovative insights and products.

